# Two Amphioxus ApeC-Containing Proteins Bind to Microbes and Inhibit the TRAF6 Pathway

**DOI:** 10.3389/fimmu.2021.715245

**Published:** 2021-07-30

**Authors:** Jin Li, Yuhui Li, Zhaoyu Fan, Shenghui Chen, Xinyu Yan, Zirui Yue, Guangrui Huang, Shumin Liu, Hao Zhang, Shangwu Chen, Meiling Dong, Anlong Xu, Shengfeng Huang

**Affiliations:** ^1^Guangdong Province Key Laboratory of Pharmaceutical Functional Genes, State Key Laboratory of Biocontrol, School of Life Sciences, Sun Yat-sen University, Guangzhou, China; ^2^Southern Marine Science and Engineering Guangdong Laboratory (Zhuhai), Zhuhai, China; ^3^Laboratory for Marine Biology and Biotechnology, Qingdao National Laboratory for Marine Science and Technology, Qingdao, China; ^4^School of Life Sciences, Beijing University of Chinese Medicine, Beijing, China

**Keywords:** ApeC, ACP, microbial binding, PGN, NF-κB, TRAF6, Amphioxus (*Branchiostoma floridae*)

## Abstract

The apextrin C-terminal (ApeC) domain is a class of newly discovered protein domains with an origin dating back to prokaryotes. ApeC-containing proteins (ACPs) have been found in various marine and aquatic invertebrates, but their functions and the underlying mechanisms are largely unknown. Early studies suggested that amphioxus ACP1 and ACP2 bind to bacterial cell walls and have a role in immunity. Here we identified another two amphioxus ACPs (ACP3 and ACP5), which belong to the same phylogenetic clade with ACP1/2, but show distinct expression patterns and sequence divergence (40-50% sequence identities). Both ACP3 and ACP5 were mainly expressed in the intestine and hepatic cecum, and could be up-regulated after bacterial challenge. Both prokaryotic-expressed recombinant ACP3 and ACP5 could bind with several species of bacteria and yeasts, showing agglutinating activity but no microbicidal activity. ELISA assays suggested that their ApeC domains could interact with peptidoglycan (PGN), but not with lipoteichoic acid (LTA), lipopolysaccharides (LPS) and zymosan A. Furthermore, they can only bind to Lys-type PGN from *Staphylococcus aureus*, but not to DAP-type PGN from *Bacillus subtilis* and not to moieties of PGN such as MDPs, NAMs and NAGs. This recognition spectrum is different from that of ACP1/2. We also found that when expressed in mammalian cells, ACP3 could interact with TRAF6 *via* a conserved non-ApeC region, which inhibited the ubiquitination of TRAF6 and hence suppressed downstream NF-κB activation. This work helped define a novel subfamily of ACPs, which have conserved structures, and have related yet diversified molecular functions. Its members have dual roles, with ApeC as a lectin and a conserved unknown region as a signal transduction regulator. These findings expand our understanding of the ACP functions and may guide future research on the role of ACPs in different animal clades.

## Introduction

The apextrin C-terminal (ApeC) is a new class of protein domains, characterized by a sequence of about 200 amino acid residues, comprising eight conserved Cysteine residues and three relatively conserved DXED motifs (1). Collectively, here we refer to the proteins containing ApeC domains as ACP (ApeC-containing protein) (2). Comparative genomic analyses show that ACPs exhibit diverse architectures and are widely distributed in* *many invertebrates. Most ACPs are present in aquatic invertebrates or invertebrates from moist environments, including cnidarians, mollusks, echinoderms, cephalochordates, flatworms, water bears, nematodes and annelids. However, there is no ACPs found in vertebrates and in major arthropod lineages (e.g. insects and crustaceans) except arachnids (2). Distant ApeC homologs were also found in bacteria, hence the origin of ApeC could be traced back to the prokaryotes. Despite their wide distribution, no ACP orthologs could be found between any phyla or sub-phyla, suggesting that ACPs obviously underwent rapid turnover and diversification.

The animal ApeC domain has eight conserved cysteines, while the bacterial ApeC-like domain has only four of them. In bacteria, ApeC-like seems to exist as single-domain proteins. In animals, ApeC actively participated in domain shuffling, which gave rise to many novel domain architectures. So far, more than twenty different domain architectures involving ApeC have been identified, suggesting great architectural diversity. This is reminiscent of other versatile domains like immunoglobulin (IG) and C-type lectin (CLECT), which are capable of exerting different functions in various domain architectures (3, 4).

The first ACP was discovered in the sea urchin *Heliocidaris erythrogramma* (5–7), which is a secreted protein with a MACPF-ApeC domain architecture. This protein is concentrated in the apical extracellular matrix in the columnar cells of the larval ectoderm, hence it was named apextrin. This apextrin is present in eggs in a type of secretory vesicles and this maternal pool, and after fertilization it will be gradually secreted to form the extracellular matrix. It is proposed that this apextrin is involved in apical cell adhesion and that its high level of expression may be necessary for strengthening the large *H. erythrogramma* embryo. Another two ACPs from the mussel *Mytilus galloprovincialis* may also have a role in embryogenesis (8): they were expressed rapidly after fertilization (up by thousands of times), and the expression decreased in later development but remained in a level in the adult stage. In Pacific oysters, the expression of an ACP was significantly up-regulated under hypoxic condition, suggesting that it may be involved in anti-stress responses (9). There are also reports suggest that ACPs may have immune functions in different species. In oysters, sea urchins and amphioxus, the expression of ACP genes could be significantly up-regulated after bacterial stimulation (10–12). A mussel ACP was highly expressed in gills and blood cells and could be further up-regulated after pathogen invasion (8, 13).

Recently, two ACPs (bjACP1 and bjACP2) from the amphioxus *Branchiostoma japonicum* have been functionally characterized (1). They were concentrated in gill and skin, and could be dramatically upregulated during acute antibacterial responses. Both them can aggregate bacteria by using their ApeC domain to bind with the cell wall component peptidoglycan (PGN). Further analysis indicates that they may interact with the muramyl dipeptide (MDP), the minimal bioactive motif of PGN. In addition to being a pattern-recognition protein, bjACP2 could regulate the TRAF6-NF-κB pathway when present in cytosol.

Previous studies have shown that some ApeC domains have carbohydrate binding capacity, and some ACPs are involved in embryonic development and host defense. However, the functions of most ACPs and their ApeC domains still remain unknown. Amphioxus (cephalochordate) is a marine chordate invertebrate, which represents the basal living chordate lineage and is therefore considered as an important proxy to study the evolution from invertebrates to vertebrates (14–19). Amphioxus has more than twenty ACP genes, hence it can be served as a valuable model to understand the functions and evolution of ACPs (2). In this study, we characterized another two ACPs (bfACP3 and bfACP5) from another amphioxus species (*Branchiostoma floridae*), which provides *a new line* of mechanistic evidence for the immune functions of ACPs.

## Materials and Methods

### Animals and Cells

Adult amphioxus (*Branchiostoma floridae*) were obtained from GL’s lab in Xiamen University (China), which derives from a stock maintained by Jr-Kai Yu originating from Tampa, Florida. The culture was maintained under previously described conditions (20), cultured in aquaria with aeration and supplied with fresh seawater (1.9%–2.9% salinity) at 20-25°C and fed with *Oocystis sp*. daily. HEK293T and Hela cell lines from ATCC, were grown in DMEM (Gibco) supplemented with 10% FCS at 37°C under 5% CO_2_.

### RNA Isolation and cDNA Synthesis

Total RNA was extracted using TRIzol Reagent (Invitrogen) and isopropanol was used for precipitation. RNA quality was determined by agarose gel electrophoresis and spectrophotometer. The isolated RNA was reverse-transcribed to synthesize the first strand cDNA using PrimeScript first Strand cDNA Synthesis Kit (Takara) using the oligo d(T) primer according to the manufacturer’s protocol. The cDNA was stored at -80°C.

### Cloning of B. floridae ACP3, ACP5, MyD88 and TRAF6

Amphioxus ACP genes were identified in the *B. floridae* genome from the Joint Genome Institute (JGI, http://genome.jgi-psf.org/Brafl1/Brafl1.home.html) database in our previous work (2). Using these sequences as baits, we blasted the transcript database of *B. floridae* (http://genome.bucm.edu.cn/lancelet/index.php) and obtained the consensus sequences of *B. floridae ACP3* (*bfACP3*, with gene ID of 102545) and *B. floridae ACP5* (*bfACP5*, with gene ID of 95439). Using human MyD88 and TRAF6 as baits, we blasted the *B. floridae* genome from the National Center for Biotechnology Information database (NCBI, http://www.ncbi.nlm.nih.gov/genbank/) and obtained the consensus sequences of *B. floridae MyD88* (*bfMyD88*, with gene ID of 118403304) and *B. floridae TRAF6* (b*fTRAF6*, with gene ID of 118414689). To obtain complete cDNA sequences, four pairs of gene-specific primers were designed for each. The amplified fragments were cloned into the pGEX-Teasy vector (Promega) and verified by sequencing at The Beijing Genomics Institute (BGI). The primers were shown in **Table 1**. These sequences have been submitted to the NCBI database under the accession number MZ004941, MZ004942, MZ442381, and MZ442382 respectively.

**Table 1:** Primers used for PCR amplification.

Primers	Primer sequence (5′ to 3′)
Gene-specific primers	
bfACP3-F	5’-ATGTTAGCACTCAAGCTCATT-3’
bfACP3-R	5’-TCACTAGGCAGGCTGGTAGTA-3’
bfACP5-F	5’-AACAGGTGTGGAGTTAAAGGTG-3’
bfACP5-R	5’-TGTGTAGGCAGCATTACGA-3’
bfMyD88-F	5’-GCAAGAATCCAGCCTTTGATCTG-3’
bfMyD88-R	5’-AAGACTGCAACGGAGGCTAA -3’
bfTRAF6-F	5’-TCTTTCCCTGTCTTCATACTTT-3’
bfTRAF6-R	5’-ATGAAACTCAGAAGTTTTTCTGTG-3’
Primers for Q-PCR	
gapdh-F	5’-CAAGGCTGTAGGCAAGGTCAT-3’
gapdh-R	5’-CTTCTTCAGTCGGCAGGTCAG-3’
bfACP1-qF	5’-CTTCGGAAGAAACAACAT-3’
bfACP1-qR	5’-ATCTTCATCGTCCCAATA-3’
bfACP2-qF	5’-GACGATAATAGCAACCAAT-3’
bfACP2-qR	5’-GTTTCCCTTCTTAAAGATACA-3’
bfACP3-qF	5’-AGTGATGGCTCCATTTAC-3’
bfACP3-qR	5’-ATCGTTGTAGGTATTCTCAT-3’
bfACP5-qF	5’-TCCTACAGAACACCGATA-3’
bfACP5-qR	5’-CTAGCTCGTTATGGTTGA-3’
Primers for Recombinant proteins	
bfACP3-32aF	5’-gccatggctgatatcggatccGACAATTTTGAGAAAACTCCAGTGG-3’
bfACP3-32aR	5’-acggagctcgaattcggatccgcGGCAGGCTGGTAGTAGCAGTACC-3’
bfACP5-32aF	5’-gccatggctgatatcggatccGACAATGTCTGTGGCGATGATC-3’
bfACP5-32aR	5’-acggagctcgaattcggatccgcTTCATCCCGTTGGTAGAAGCA-3’
Primers for construction of expression vector	
bfACP3-HA-F	5’-actactggtacctctggatccATGTTAGCACTCAAGCTCATTGTGC-3’
bfACP3-HA-R	5’-cttaccgaattctgtggatccGGCAGGCTGGTAGTAGCAGTACC-3’
Myc-bfACP3-F	5’-tggccatggaggcccgaattcggATGTTAGCACTCAAGCTCATTGTGC-3’
Myc-bfACP3-R	5’-gatccccgcggccgcggtaccCTAGGCAGGCTGGTAGTAGCAGT-3’
Flag-bfMyD88-F	5’-gacgatgacaagggcggtaccATGGCAACAAACGCGCCA-3’
Flag-bfMyD88-R	5’-ttctgtggatccagaggtaccTCACGGGCGAGAGAGGGC-3’
Flag-bfTRAF6-F	5’-gacgatgacaagggcggtaccATGAAGCCAGGAGGGAGGG-3’
Flag-bfTRAF6-R	5’-ttctgtggatccagaggtaccCTATTGTGGCTGCACCGTACAT-3’
bfACP3-24-561-F	5’-accgagatctctcgaggtaccGACAATTTTGAGAAAACTCCAGTGG-3’
bfACP3-24-561-R	5’-gatccccgcggccgcggtaccCTAGGCAGGCTGGTAGTAGCAGT-3’
bfACP3-24-204-F	5’-accgagatctctcgaggtaccGACAATTTTGAGAAAACTCCAGTGG-3’
bfACP3-24-204-R	5’-gatccccgcggccgcggtaccCTACCCCACCTCCACTGCCG-3’
bfACP3-205-561-F	5’-accgagatctctcgaggtaccAATCAGTGGATGGAGGGCG-3’
bfACP3-205-561-R	5’-gatccccgcggccgcggtaccCTAGGCAGGCTGGTAGTAGCAGT-3’
bfACP3-205-358-F	5’-accgagatctctcgaggtaccAATCAGTGGATGGAGGGCG-3’
bfACP3-205-358-R	5’-gatccccgcggccgcggtaccCTATTTTTTCACATGTGGCTCCAG-3’
bfACP3-165-358-F	5’-accgagatctctcgaggtaccGGCACCAGCAGTGACCAACA-3’
bfACP3-165-358-R	5’-gatccccgcggccgcggtaccCTATTTTTTCACATGTGGCTCCAG-3’
bfACP3-124-358-F	5’-accgagatctctcgaggtaccAAGGAAACAGGCAACGAGAACG-3’
bfACP3-124-358-R	5’-gatccccgcggccgcggtaccCTATTTTTTCACATGTGGCTCCAG-3’
bfACP3-24-358-F	5’-accgagatctctcgaggtaccGACAATTTTGAGAAAACTCCAGTGG-3’
bfACP3-24-358-R	5’-gatccccgcggccgcggtaccCTATTTTTTCACATGTGGCTCCAG-3’
bfACP3-359-561-F	5’-accgagatctctcgaggtaccTGGCCTACTGGAACCTATGGC-3’
bfACP3-359-561-R	5’-gatccccgcggccgcggtaccCTAGGCAGGCTGGTAGTAGCAGT-3’

### Bioinformatic Analysis

The domain structure was predicted on the SMART Website (http://smart.embl-heidelberg.de). BLASTP was performed to analyze the sequence identities. The isoelectric point (pI) and m.w. were estimated on ExPASy Website (http://www.expasy.org/tools/). Multiple sequence alignments were analyzed using MEGA-X (21) by using the ClustalW algorithm and were manually corrected using GeneDoc software (22). The Neighbor-joining phylogenetic tree was built using MEGA-X (21) with the JTT matrix-based method (23), 1000 bootstrap tests and pairwise deletion of gaps. The Maximum likelihood phylogenetic tree was built using MEGA-X with the WAG model, Gamma distribution of rates across sites model and 1000 bootstrap tests (24).

### Real-Time Quantitative RT-PCR

Real-time quantitative RT-PCR (Q-PCR) was performed and analyzed as described (25). For tissue expression profiles, five healthy adults were chosen and not fed for 3 days to empty the gut. Then different tissues (muscle, skin, gill, ovary, hepatic cecum, intestine) were dissected under an optical microscope. Total RNA was extracted and reversely transcribed to the first strand cDNA according to the method in 2.2. Q-PCR was performed on the Roche LightCycler 480 instrument (using the 384-well module). SYBR^®^ Green Realtime PCR Master Mix (Toyobo) was used for the assays according to the manufacturer’s protocol. The reaction volume was 10µl, with 40ng 1st-strand cDNA and a primer concentration of 0.5 µM. The PCR program is 95 °C for 3 min, followed by 40 cycles of 95 °C for 15 s, 60 °C for 30 s, and 72 °C for 20 s. Reaction of each sample was performed in triplet. Amphioxus *gapdh* was used as the internal control. The cycle threshold values were calculated by the Roche LightCycler 480 software. Data were quantified using the 2^-ΔΔCt^ method based on the cycle threshold values. All results were confirmed by repeating the assays by one or two more times. Primers used for Q-PCR were listed in **Table 1**.

For monitoring the gene expression changes after immune stimulation, ten adults were collected for each treatment and not fed for 24h in filtered seawater. Each amphioxus was injected into the coelom with 20 ul LPS (1 mg/mL in PBS, from *E. coli* O111:B4, Sigma), LTA (1 mg/mL in PBS, from *S. aureus*, Sigma), inactivated bacteria or PBS (as control). The bacteria were 1:1 mixture (vol/vol) of formalin-inactivated *Staphylococcus* aureus (2×10^8^ cells/mL) and *Vibrio anguillarum* (2×10^8^ cells/mL). Then, the gut (hepatic cecum and intestine) and the gill were harvested at 0, 2, 4, 8, 12, 24, and 48h after injection and immediately frozen using liquid nitrogen.

### Preparation of Recombinant Proteins

To obtain soluble proteins, we expressed recombinant ACP proteins using the pET32a system, a thioredoxin (TRX) fusion system containing a 6×His tag to facilitate the purification on a Ni^2^-chelating Sepharose column and a partner TRX to help the proteins fold correctly. The ClonExpress^®^II One Step Cloning Kit (Vazyme) was used to insert the coding regions of mature bfACP3 (Asp24 to Ala561) and bfACP5(Asp21 to Glu636) into the pET32a plasmids. The plasmids were transformed into *E. coli* BL21 (DE3). The transformed bacteria were cultured to an OD=0.6-0.8 and induced with 1 mM IPTG at 37°C for four hours. After induction, the bacterial cells were collected and sonicated for lysis. The cell lysis supernatant was purified through a Ni^2^-chelating Sepharose column (GE Healthcare). The recombinant proteins were eluted with 250 mM imidazole, dialyzed in PBS buffer at 4°C for 12 hours three times and concentrated by ultrafiltration using an Ultrafree centrifugal filter device (Millipore). Pierce™ BCA Protein Assay Kit (Thermo) was used to determine the protein concentration according to the manufacturer’s protocol. Primers used for preparation of recombinant proteins were listed in **Table 1**.

### Microbial Binding Assays

Bacteria Staphylococcus aureus, Enterococcus faecalis, Escherichia coli, Vibrio anguillarum, Vibrio parahemolyticus, Acinetobacter calcoaceticus and Saccharomyces cerevisiae were respectively inoculated into suitable liquid medium and cultivated overnight under suitable conditions. V. anguillarum was cultured with high-salt Luria-Bertani (LB) medium at 28°C, V. parahemolyticus was cultured with seawater medium at 28°C, S. cerevisiae was cultured with YPD medium at 28°C, and the remaining bacteria were cultured with LB medium at 37°C. The next day, the cells were collected and washed with PBS buffer and resuspended in PBS. Approximately 2×10^6^ microbes were incubated with 1 μg of purified recombinant proteins in 1 mL PBS at 4°C overnight with gentle orbital rotation. Microbes were centrifuged at 12,000 × g for 1 min at 4°C and the pellets were washed five times with 1 mL of PBST (0.05% Tween-20 in PBS). The washed pellets were then suspended in 100μl PBS and 20μl 6×loading buffer and boiled at 100°C for 10 min. Western blot was performed with an anti-His mouse monoclonal antibody (Sigma) to validate the binding proteins.

### Microbial Aggregation Assays

Microbes collected from liquid cultures were suspended in 1 mL PBS and mixed with 50μl Fluorescein isothiocyanate (FITC) (sigma, 10 mg/mL in DMSO). The reaction was incubated at room temperature in the dark for 3h with gentle agitation. Then the microbes were washed five times and resuspended in PBS. 10μg TRX fusion proteins were incubated with 50 μl FITC-labeled *S. aure*us (2 × 10^8^ cells/mL), *E. coli* (2 × 10^8^ cells/mL), *V. parahemolyticus* (2 × 10^8^ cells/mL) or *S. cerevisiae* (2 × 10^7^ cells/mL) at room temperature in the dark for 2h, respectively. The agglutinating reaction was examined immediately under fluorescence microscopy (Carl Zeiss).

### Binding Assays of ACPs With Microbial Cell Wall Components

ELISA was used to analyze the binding of ACP proteins with soluble microbial cell wall components as previously described (26). In brief, 20 μg of lipopolysaccharide (LPS, from *E. coli*, Sigma), lipoteichoic acid (LTA, from *S. aureus*, Sigma), peptidoglycan (PGN, from *S. aureus* or *Bacillus subtilis*, Sigma), D-Mannose (Sigma), zymosan A (from *S. cerevisiae* Sigma), muramyl dipeptide (MDP, Sigma), GlcNAc (NAG, Sigma) and MurNAc (NAM, Sigma) were used to coat 96-well microplate (Corning 96-well Clear Polystyrene High-Bind Strip well Microplate) at 37°C for 3 h (PGN and zymosan A are ultrasonically solubilized). After washing with PBS three times, the wells were blocked with PBST (0.05% Tween 20 in PBS) containing 10% (wt/vol) skim milk overnight at 4°C. Several concentrations of ACP proteins were added to the well and incubated at 37°C for 2 h. Binding proteins were detected with mouse anti-His mAb (sigma, diluted 10000-fold) at room temperature for 1h, followed by an hour incubation with HRP-labeled anti-mouse Secondary antibodies (Abmart, diluted 5000-fold). Incubating plate with 100ul TMB Substrate Solution (Thermo) at room temperature for 15 minutes and stop reaction by adding 50µL of 2M sulfuric acid. The absorbance was read at 450 nm immediately. Reaction of each sample was performed in triplet and the assay was repeated at least three times. The insoluble PGN-binding activities of ACP proteins were detected by pull-down assays as previously described (26). In brief, various concentrations of insoluble PGNs were incubated with 5 μg of ACP proteins in 1 mL PBS at 4°C for 1 h. The samples were centrifuged at 15,000 × g for 10 min and pellets were washed with PBST (0.05% Tween 20 in PBS) four times, suspended in 100 μl PBS and 20 μl 6×loading buffer, and then boiled at 100°C for 10 min. Western blot analysis of the binding proteins was performed using mouse anti-His mAb (sigma).

### Antimicrobial Activity Assays

The growth curves of *Staphylococcus aureus* and *Vibrio parahemolyticus* cultured with recombinant ACP proteins were tested as follows. Two single colonies were picked up separately and transferred into 1 mL of LB or Sea water broth. A volume of 50 μl of cell suspension was mixed with purified recombinant proteins and added to 1 mL broth. *S. aureus* was incubated at 200 rpm at 37°C and *V. parahemolyticus* was incubated at 200 rpm at 28°C. OD_600_ of each sample was measured every 1 h. The Oxford cup method was performed on a petri dish. Twenty milliliters of warm nutrient agar (1.5%) were poured into a 90-mm plate and cooled to form the base medium. Ten milliliters of 0.8% warm nutrient agar mixed with *S. aureus* or *V. parahemolyticus* were poured onto the base medium and then quickly placed the Oxford cup and gently press. Then, the targeted protein or antibiotic in 100 μL of PBS was added to the pores at a final concentration of 1 μg/μL. The plates were incubated at a suitable temperature for 16h or 40 h. A transparent ring around the pores indicated antibacterial activity.

### Construction of the Expression Vectors

For the study of the subcellular localization and coimmunoprecipitation (Co-IP) test between bfACP3 and bfTRAF6, full-length bfACP3 was inserted into the pcDNA3.0 vector (Clontech) with a C-terminal HA Tag (transformed by our laboratory, the HA coding sequence was inserted after the *XbaI* restriction site) and bfTRAF6 was fused with Flag tag and inserted into the pcDNA 3.0 vector (Clontech, transformed by our laboratory, the Flag coding sequence was inserted in front of the *Kpn I* restriction site). For the expression of the truncated mutants of bfACP3, PCR fragments encoding amino acids 24-561, 24-204, 205-561, 205-358, 165-358, 124-358, 24-358 and 359-561 were fused with myc tag, and inserted into the expression plasmid pCMV-Myc vector (Clontech). For the reporter assays and ubiquitination experiment, full-length bfACP3 was cloned into the pCMV-Myc vector (Clontech) and bfMyD88 was constructed in the same way as bfTRAF6. The full-length sequences of bfMyD88 and bfTRAF6 are shown in the **Supplementary Figures 6** and **7**, respectively. The ClonExpress^®^ II Kit (Vazyme) was used for the construction of recombinant expression vectors. The vectors were verified by sequencing and the expression of proteins were confirmed by western blot. Primers were described in **Table 1**.

### Luciferase Reporter Assays

HEK293T cells were digested by trypsin and seeded in 48-well plates. After 24h, the cells were transfected with equivalent mixed expression plasmids as previously descripted (27), which consist of the indicated amount of expression vectors, 50 ng per well of the NF-κB response promoter luciferase reporter plasmid pNF-κB-Luc (StrataGene), and 5 ng *Renilla* luciferase reporter plasmid pRLTK (Promega) per well to normalize the data due to different transfection efficiency between wells, and empty vectors to complement the total plasmid quantity of each well to the same. After 24 h, HEK293T cells were harvested and measured by dual-luciferase reporter assay system (Promega). Each experiment was performed in triplicate and was repeated at least three times in all cases. The data is shown as the fold change relative to that measured in cells transfected with empty vector.

### Western Blot and Co-Immunoprecipitation (Co-IP) Assays

Western blot and Co-IP assays were performed to demonstrate the interaction of amphioxus bfACP3 and bfTRAF6. HEK293T cells in six-well dishes were transfected with 6μg constructed plasmid (3 μg/each expression vector) after 24 h seeded. At 24h post-transfection, whole-cell extracts were prepared in IP lysis buffer (Cell Signaling Technology) supplemented with cocktail protease inhibitor (Roche) on ice. Then the cell lysates were fractionated by 10% SDS-PAGE and then transferred onto a nitrocellulose filter membrane. The membrane was blocked with 5% skim milk in PBST (0.05%Tween-20 in PBS) at room temperature for 1 h and then incubated with the corresponding primary antibody (Sigma) at 4°C overnight. Wash the blot in PBST three times and incubated with HRP-conjugated Goat Anti-Mouse IgG (Abmart) at room temperature for 1 h and detected with enhanced chemiluminescence reagent (Millipore, USA). For Co-IP assays, the cell lysates were incubated with 1μg primary antibodies (Sigma) at 4°C overnight then incubated with Protein G Sepharose (Roche) at 4°C for 4-6 h. The mixture was washed three times with cell lysis buffer and analyzed by Western blot.

### Subcellular Localization Analysis

Hela cells were seeded on coverslips (10 mm×10 mm) in a 24-well plate. After 24 h, cells were transfected with 2μg indicated expression plasmids (1 μg/each expression vector) by Lipofectamine 3000 (Thermo) according to the manufacturer’s instructions. After 24 h since transfection, cells we washed twice in PBS, fixed in 4% paraformaldehyde solution, and permeabilized by 0.1% Triton X-100 in PBST (0.05%Tween-20 in PBS). After washing with PBST three times, the wells were blocked with PBST containing 10% (wt/vol) BSA at room temperature for 1 h. Cells were further incubated with primary mAb (Sigma) at 4°C overnight, washed three times in PBST, and incubated with the second antibody Alexa Fluor 568 Goat anti-rabbit IgG (Invitrogene) or Alexa Fluor 488 Goat anti-mouse IgG (Invitrogene) for 1 h. Following triple washing in PBS, cells were stained with 0.2 μg/mL DAPI for 5min. The slips were washed three times in PBS, mounted in ProLong™ Glass Antifade Mountant (Thermo) and photographed with a Carl Zeiss Axio Imager Z1 fluorescence microscope.

### Statistical Analysis

Student’s t-tests were performed to compare the means of three sample groups. In all cases, differences of p<0.05 were considered significant. *p < 0.05, **p < 0.01, ***p < 0.001 indicate statistical differences. All experiments were repeated at least three times.

## Results

### Cloning and Sequence Analysis of Amphioxus ACP3 and ACP5

**Figure 1:**
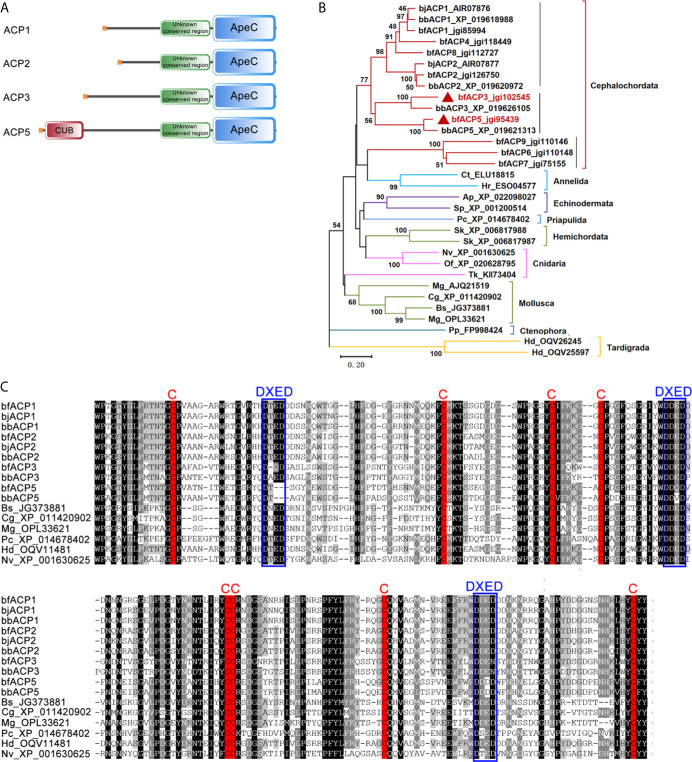
Comparison of the amino acid sequences of bfACP3 and bfACP5 to other ACPs. **(A)** The domain architectures of amphioxus ACP3 and ACP5 compared with ACP1 and ACP2. **(B)** The phylogenetic tree of representative ACPs from different aquatic animal phyla based on the sequence of ApeC domains. The tree was constructed using the neighbor-joining method. Numbers on the lines indicate the percentage bootstrap values for 1000 replicates. The tree is drawn to scale, with branch lengths in the same units as those of the evolutionary distances used to infer the phylogenetic tree. A Maximum Likelihood tree based on the same alignment is provided in **Supplementary Figure 4** and a more detailed version is shown in **Supplementary Figure 5**. **(C)** Multiple alignment of the ApeC domains of ACPs from amphioxus and other aquatic species. The conserved Cysteine residues and DXED motifs are marked with red and blue boxes, respectively. bj, *Branchiostoma japonicum*; bf, *Branchiostoma floridae*; bb, *Branchiostoma belcheri*; Bs*, Botryllus schlosseri*; Cg, *Crossostrea gigas*; Mg, *Mytilus galloprovincialis*; Pc, *Priapulus caudatus*; Hd, *Hypsibius dujardini*; Nv, *Nematostella vectensis*; Ap, *Acanthaster planci*; Sp, *Strongylocentrotus purpuratus*; Sk, *Saccoglossus kowalevskii*; Tk, *Thelohanellus kitauei*; Of, *Orbicella faveolata*; Ct, *Capitella teleta*; Hr, *Helobdella robusta*; Pp, *Pleurobrachia pileus*.

We previously identified 21 ACP gene models from the genome and transcriptomes of the amphioxus *Branchiostoma floridae* (bf) (2). In this study, we cloned the full-length cDNA sequences for bfACP3 and bfACP5, which encode a 561aa protein and a 636aa protein, respectively (**Supplementary Figures 1** and **2**). ACP3 has a signal peptide, an unknown conserved region (**Supplementary Figure 3**) and a C-terminal ApeC domain. ACP5 structurally resembles ACP3, but has a complement-related CUB domain right behind the signal peptide (**Figure 1A**). Phylogenetic analysis based on the ApeC domains suggests that ACP3 and ACP5 are close paralogs to the previously-reported ACP1 and ACP2 (**Figure 1B**, **Supplementary Figures 4** and **5**). But differences are also obvious. For example, ACP3/ACP5 have a longer middle spacer region than ACP1/ACP2. ACP5 even has a CUB domain (**Figure 1A**). The CUB domain (for complement C1r/C1s, Uegf, Bmp1) is almost exclusively found in extracellular matrix or plasma proteins (28). Based on the multiple alignment of the ApeC domain, ACP3 shared only 50% sequence identity with ACP1 and 47% with ACP2, while ACP5 shared only 47% sequence identity with ACP1 and 46% with ACP2. The sequence identity decreases in the region outside the ApeC domain. A typical ApeC domain has eight conserved cysteines and three DXED motifs. The ApeC domains of amphioxus ACP1 and ACP2, as well as many other invertebrate ACPs, preserve all these conserved features. Although ACP3 and ACP5 preserve all eight cysteines, ACP3 has mutations in the first and third DEXD motifs, and ACP5 has mutations in all three DXED motifs (**Figure 1C**). We speculate that the deviation from the canonical motif may have functional implications, but so far the function of these motifs remains unknown. Overall, amphioxus ACP3 and ACP5 have their own sequence and structural characteristics, though belonging to the same class with ACP1 and ACP2.

### The Expression Patterns of *bfACP3* and *bfACP5*

**Figure 2:**
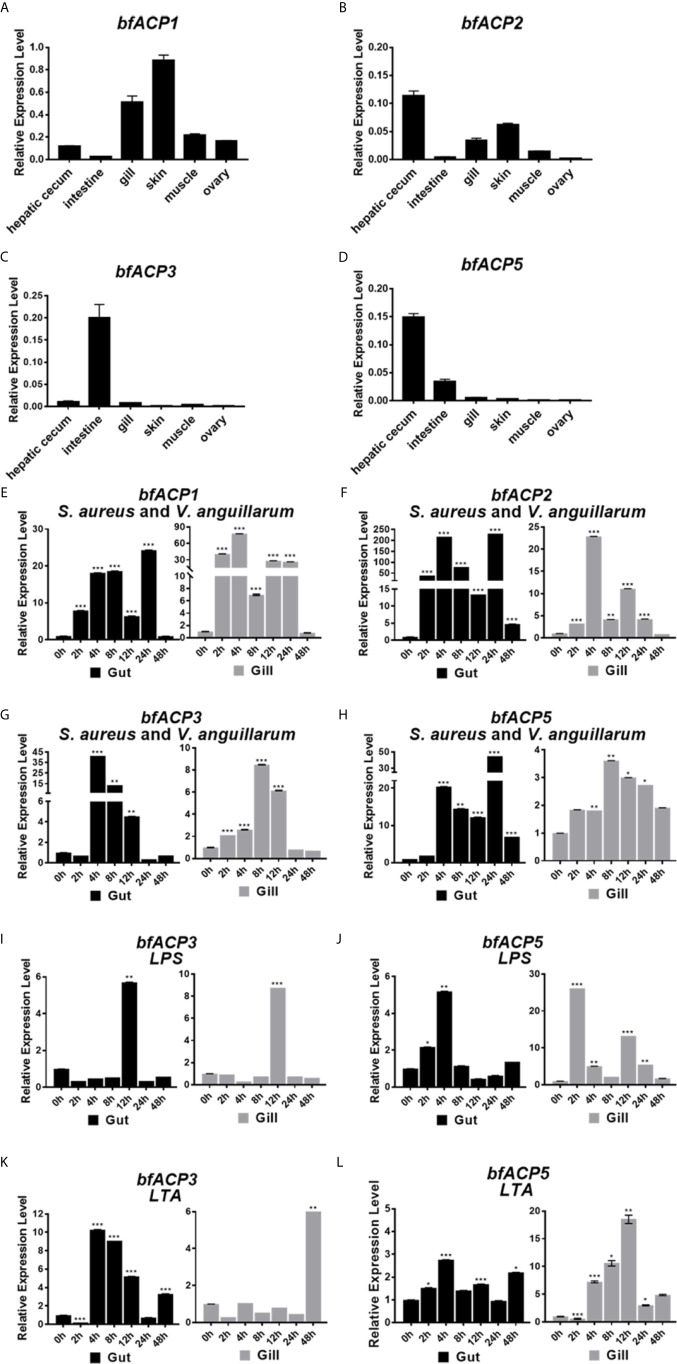
Q-PCR analysis of the expression profile of *bfACP3* and *bfACP5*. The relative level of *bfACP1* **(A)**, *bfACP2* **(B)**, *bfACP3* **(C)** and *bfACP5* **(D)** mRNA in different tissues, respectively. Experiments were performed with five amphioxus. Data were expressed as a ratio to the *gapdh* mRNA expression and were plotted as the mean ± SD. **(E–H)** The relative expression level of *ACP* mRNA in gut (hepatic cecum and intestine) and gill after challenge with mixed inactivated bacteria (*S. aureus* and *V. anguillarum* in 1:1 ratio). **(I–L)** The relative expression level of *bfACP3 and bfACP5* mRNA in gut (hepatic cecum and intestine) and gill after challenge with Gram-negative cell wall component LPS (1mg/mL) and Gram-positive bacteria cell wall component LTA (1mg/mL) for different time. Data were expressed as a ratio to the ACP mRNA expression level of unchallenged naive animals and were normalized to the *gapdh* expression. All the samples were analyzed in three replicates and mean ± SD is plotted, *p < 0.05, **p < 0.01, ***p < 0.001. One representative experiment out of three is shown.

Realtime quantitative RT-PCR analyses showed that *bfACP1* and *bfACP2* had substantial expressions in at least four of six examined tissues of adult amphioxus (**Figures 2A, B** **)**. In particularly, they exhibited high expressions in the gill and skin, which are in direct contact with the environmental microbial pathogens. On the other hand, the mRNA of *bfACP3* and *bfACP5* had limited tissue distribution, with *bfACP3* predominantly expressed in the intestine and *bfACP5* predominantly in the intestine and hepatic cecum (**Figures 2C, D** **)**. After challenged with formalin-inactivated bacteria (the Gram-positive *Staphylococcus aureus* plus the Gram-negative *Vibrio anguillarum*), the mRNA of all four amphioxus ACPs could be upregulated in the gut and gill in adult amphioxus (**Figures 2E–H**). However, *bfACP3* and *bfACP5* were less responsive in the gill than in the gut, despite that their basal expression in the gill was extremely low (0.5-0.8% of the *gapdh* expression). The expression of *bfACP3* and *bfACP5* also responded to the major components (LPS & LTA) of the bacterial cell walls, but the profiles were quite complicated (**Figures 2I–L**). Taken together, while amphioxus *ACP1* and *ACP2* have substantial expressions in multiple tissues and are capable of dramatic upregulation during the acute immune response, the expression and function of *ACP3* and *ACP5* appear to mainly locate in the gut (intestine and hepatic cecum).

### BfACP3 and bfACP5 Bind and Aggregate Different Microbes

**Figure 3:**
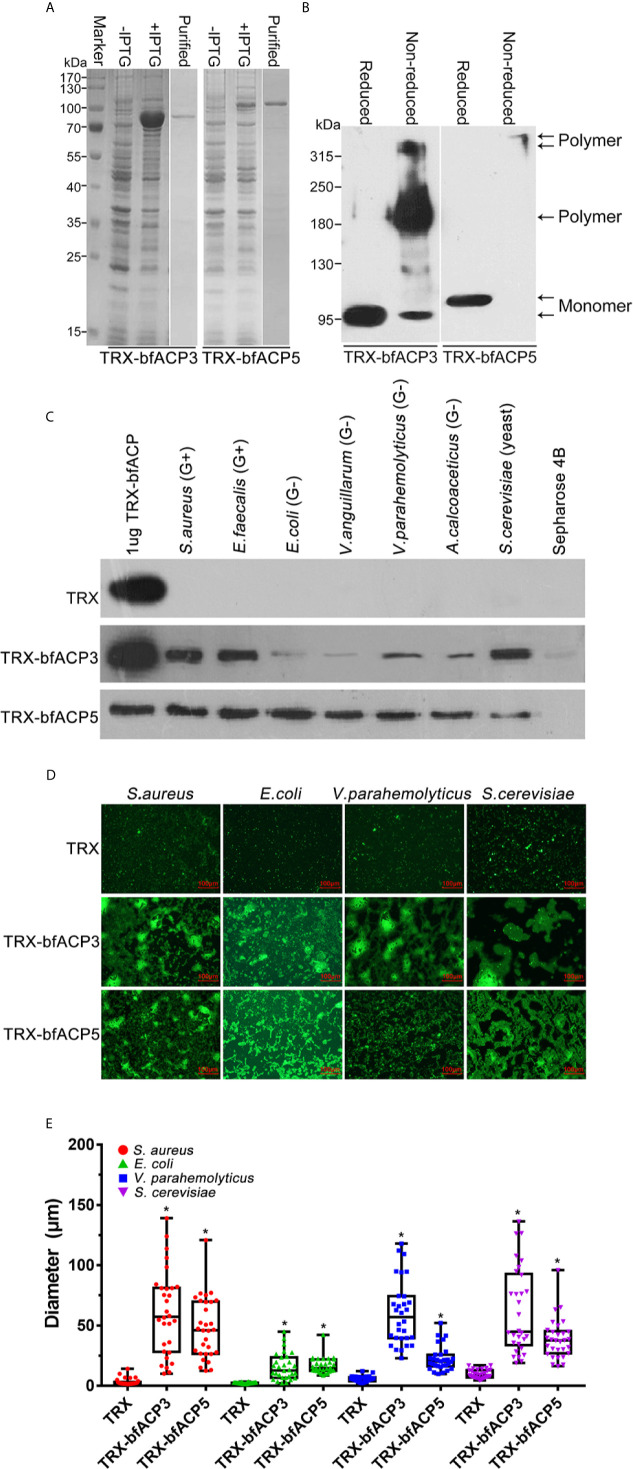
Binding and aggregation of the microbes by bfACP3 and bfACP5. **(A)** SDS-PAGE analysis of samples taken during the purification of recombinant TRX-bfACP3 and TRX-bfACP5. **(B)** Reducing and Non-reducing SDS-PAGE of ACPs. The bands corresponding to the monomer or oligomer were marked. **(C)** The binding of microorganisms by recombinant TRX-bfACP3 and TRX-bfACP5 protein. Approximately 2×10^6^ living microbes were incubated with 1μg TRX fusion proteins in 1 mL PBS at 4°C overnight and the stirringly washed pellets were subjected to the SDS-PAGE and detected by Western blot with anti-6×His monoclonal antibody. One representative experiment out of three is shown. **(D)** Aggregation of the microbes by TRX-bfACP3 and TRX-bfACP5. 10μg TRX fusion proteins were incubated with 50 μl FITC-labeled *S. aure*us (2 × 10^8^ cells/mL), *E coli* (2 × 10^8^ cells/mL), *V. parahemolyticus* (2 × 10^8^ cells/mL) or *S. cerevisiae* (2 × 10^7^ cells/mL) at room temperature in the dark for 2h, respectively. The agglutinating reaction was examined using fluorescence microscopy. **(E)** Box plot showing the diameters of green puncta in microbial aggregation assays. Box plot explanation: upper horizontal line of box, 75th percentile; lower horizontal line of box, 25th percentile; horizontal bar within box, median; upper horizontal bar outside box, maximum value; lower horizontal bar outside box, minimum value. *p < 0.05 *versus* TRX control.

Here we showed that bfACP3 and bfACP5 could be greatly upregulated in the gut by bacterial stimulation (**Figure 2**), so they might also have the capacity to bind microbes. To test this, we prepared purified recombinant His-tagged TRX-bfACP3 and TRX-bfACP5 fusion proteins (**Figure 3A**). Since bfACP3 and bfACP5 have many cysteines for potential intermolecular disulfide bonds (**Figure 1C**), we performed reduced and non-reduced SDS-PAGE assays and found that both proteins could form dimers or tetramers (**Figure 3B**). We then incubated the recombinant proteins with different microbes. The microbial pellets were assessed by western blot using anti-His monoclonal antibodies. The results showed that bfACP3 bound strongly to yeasts (*S. cerevisiae*) and Gram-positive bacteria, but bound to Gram-negative bacteria with weaker affinity (**Figure 3C**). Besides, based on the band intensity estimation, up to 50% of the bfACP3 recombination proteins in this assay bound to the microbes. As for bfACP5, it could bind with all the tested microbes (**Figure 3C**). Moreover, fluorescence microscopy showed that both bfACP3 and bfACP5 could aggregate FITC-labeled *S. aureus*, *E. coli*, *V. parahaemolyticus* and yeast *S. cerevisiae* (**Figure 3D**). Quantification of the diameter of green microbial puncta showed a significant agglutination effect (**Figure 3E**). In a previous study, ACP1 and ACP2 were shown to bind and aggregate Gram-positive bacteria but not Gram-negative bacteria (1). Therefore, the microbial binding spectrum of the amphioxus ACP3 and ACP5 seems quite different from that of ACP1 and ACP2. We wondered if such differences were related to the less conserved DEXD motifs in the ApeC domain of ACP3 and ACP5 (**Figure 1B**).

### BfACP3 and bfACP5 Have No Inhibitory Activity Against *S. aureus* and *V. parahaemolyticus*

**Figure 4:**
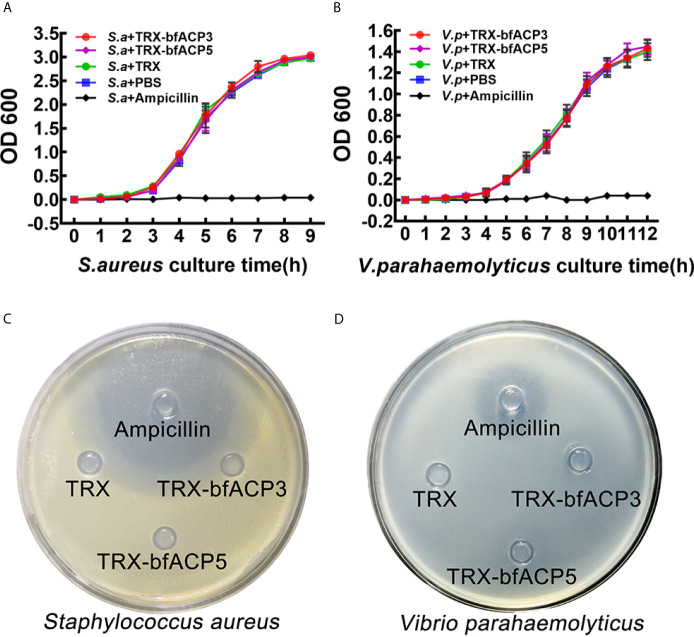
BfACP3 and bfACP5 are not antibacterial against *S. aureus* and *V. parahaemolyticus.* Growth curves of *S. aureus* **(A)** and *V. parahaemolyticus* **(B)** in the presence of TRX-bfACP3 or TRX-bfACP5 in medium while being shaken. OD_600_ was measured every 1 h after starting the culture (mean ± SD, n = 3). Oxford Cup experiments cultured with *S. aureus* **(C)** or *V. parahaemolyticus* **(D)** were performed with 100ul Ampicillin (100μg/mL), TRX (0.2μg/μl), TRX-bfACP3 (0.2μg/μl) and TRX-bfACP5 (0.2μg/μl). Then, the plates were incubated at 37°C for 16 h (*S. aureus*) or 28°C for 40 h (*V. parahaemolyticus*). A transparent ring around the cups signifies antibacterial activity.

We monitored the growth curves of *S. aureus* and *V. parahaemolyticus* in the presence of bfACP3 or bfACP5 proteins (with Ampicillin as a positive control). Both proteins had a negligible effect on the growth rates of the bacteria under these experimental conditions (**Figures 4A, B** **)**. Moreover, the Oxford cup experiments showed that TRX-bfACP3 and TRX-bfACP5 fusion proteins had no discernable inhibitive effect on *S. aureus* and *V. parahaemolyticus* (**Figures 4C, D** **)**. These results are reminiscent of a previous study, in which amphioxus ACP1 and ACP2 showed no detectable bacteriostatic or bactericidal activity against *S. aureus* (1). In conclusion, amphioxus ACP1, ACP3, ACP3 and ACP5 may all have microbial binding and aggregating activities, but have no killing activity.

### Binding Specificity of bfACP3 and bfACP5 to Microbial Cell Wall Components

**Figure 5:**
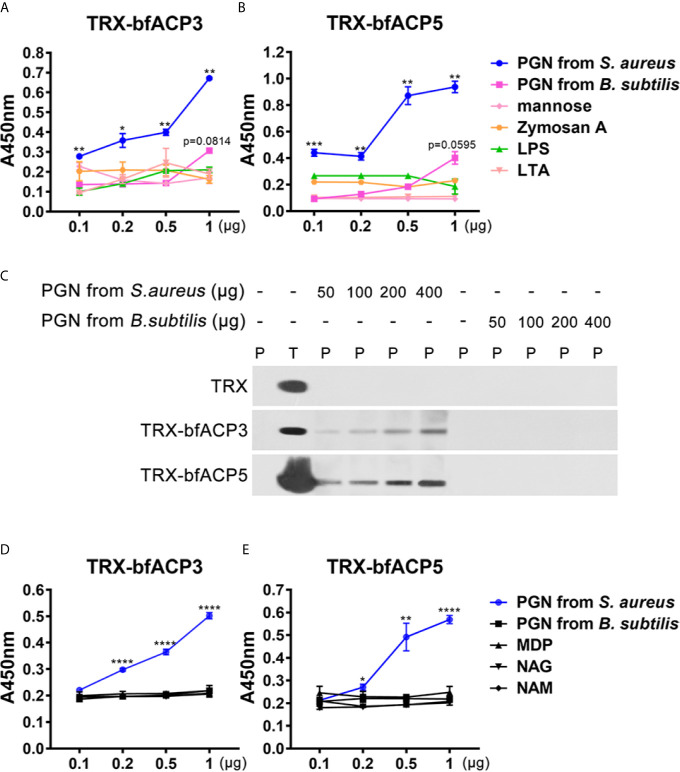
BfACP3 and bfACP5 directly interacted with the components of the microorganism cell walls. **(A, B)** ELISA analysis of the interaction between recombinant fusion TRX-bfACP3 and TRX-bfACP5 to the components, respectively. Plates were coated with 20μg components, incubated with TRX-bfACP3 or TRX-bfACP5 at 37°C overnight and detected with anti-6×His monoclonal antibody. Three biological replicates were designed for each experiment, and three technical replicates were performed, showing one of the representative results. Background absorbance with TRX was subtracted. **(C)** Pull-down analysis of the binding of 5μg recombinant fusion ACPs to PGN from *S. aureus* or *B subtilis*. P, pellet protein; T, total protein. **(D, E)** ELISA analysis of the interaction between recombinant fusion ACPs and 20μg monomers that are parts of peptidoglycan. NAG, GlcNAc; NAM, MurNAc. *p < 0.05, **p < 0.01, ***p < 0.001, ****p < 0.0001.

We performed ELISA assays to detect which microbial cell wall component was recognized by bfACP3 and bfACP5. The results indicated that both bfACP3 and bfACP5 recombinant proteins had a high binding affinity with the soluble PGN from *S. aureus*, but not with LPS, LTA, mannose, zymosan A and the PGN from *B. subtilis* (**Figures 5A, B** **)**. PGN can be classified into the DAP-type and the Lys-type according to the difference in amino acid residues and cross-linking methods (29). PGN from *S. aureus* is Lys-type and PGN from *B. subtilis* is DAP-type. Therefore, it seems that bfACP3 and bfACP5 could only recognize the Lys-type PGN. Pull-down assays confirmed that bfACP3 and bfACP5 were directly bound to insoluble PGN from *S. aureus* in a dose-dependent manner but not to PGN from *B. subtilis* (**Figure 5C**). The basic building blocks of PGN include N-acetylglucosamine (GlcNAc; NAG), N-acetylmuramic acid (MurNAc; NAM) and Muramyl dipeptide (MDP), but we detected no interaction between these motifs with our recombinant bfACP3 and bfACP5 (**Figures 5D, E** **)**. This contrasted the finding that the recombinant proteins of amphioxus ACP1 and ACP2 could bind with MDP (1). We suspect that the intact molecule or certain conformation of Lys-type PGN are required for the stable interaction with bfACP3 and bfACP5. So far, we may conclude that bfACP3 and bfACP5 could agglutinate Gram-positive bacteria by recognizing their cell-wall component PGN, though there is more to be done to explain how bfACP3/bfACP5 could bind with yeast and how bfACP5 could bind with Gram-negative bacteria.

### BfACP3 Suppressed MyD88 and TRAF6 Mediated NF-κB Activation

**Figure 6:**
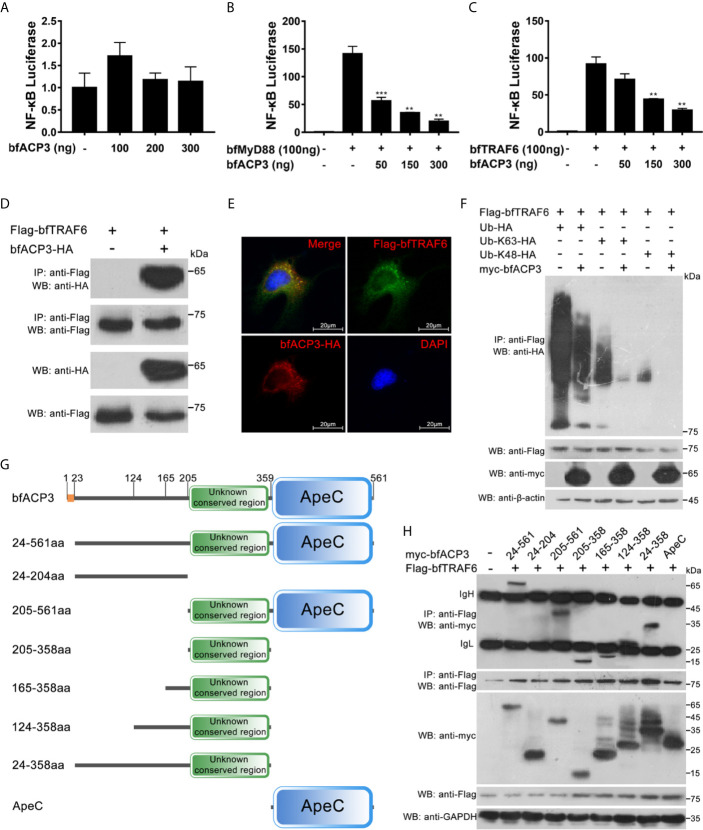
BfACP3 negatively regulated TRAF6-NF-κB pathway by suppressing the ubiquitination of bfTRAF6. For luciferase reporter assays, HEK293T cells were co-transfected with NF-κB transcriptional luciferase reporter plasmid, *Renilla* luciferase reporter plasmid, bfMyD88 or bfTRAF6 vectors, together with bfACP3 vector. For Co-IP assays and colocalization assay, HEK293T cells and Hela cells were used, respectively. **(A)** BfACP3 hardly activated NF-κB signal. **(B, C)** BfACP3 negatively regulated bfMyD88-induced **(B)** and bfTRAF6-induced **(C)** NF-κB activation. The representative results are shown as means ± SD (n=3) of three experiments. **p < 0.01, ***p < 0.001. **(D)** Co-IP assay showing that bfACP3 interacts with amphioxus bfTRAF6 when overexpressed in HEK293T cells. **(E)** Immunofluorescence analysis of the subcellular co-localization of bfTRAF6 and bfACP3. HeLa cells were co-transfected with HA-tagged bfACP3 and Flag-tagged bfTRAF6, then stained with rabbit anti-HA and mouse anti-Flag antibody, followed by incubating with the Alexa Fluor 568 goat anti-rabbit and Alexa Fluor 488 goat anti-mouse secondary antibody respectively. **(F)** Ubiquitination assays indicate that bfACP3 suppressed the polyubiquitin chains of bfTRAF6. **(G)** The full-length and truncated mutants of bfACP3 used in this study. The amino acids were numbered according to bfACP3 sequence. **(H)** Co-IP assay between bfACP3 mutants and bfTRAF6 indicated that the unknown conserved non-ApeC region of bfACP3 is responsible for the interaction with bfTRAF6.

It was shown that when retained in cytoplasm, amphioxus ACP2 could interact with TRAF6 and suppress TRAF6 mediated NF-κB activation (1). We wondered if bfACP3 has similar intracellular functions. We transfected the HEK293T cells with the NF-κB luciferase reporter plasmid and the bfACP3 expression plasmid, together with the bfMyD88 or bfTRAF6 expression plasmids. Luciferase reporter assays showed that bfACP3 alone could not affect the NF-κB signal, but could inhibit the NF-κB signal activated by bfMyD88 and bfTRAF6 in a dose-dependent form (**Figures 6A–C**). Co-IP assays using 293T cells showed that bfACP3 could physically interact with bfTRAF6 (but not with bfMyD88) in our experimental condition (**Figure 6D**). Immunofluorescence imaging analysis using HeLa cells confirmed that bfACP3 and bfTRAF6 could co-localize in the same subcellular punctate structures (**Figure 6E**). These results suggested that when presented in cytoplasm, bfACP3 could negatively regulate the MyD88-TRAF6-NF-κB pathway by interacting with bfTRAF6.

### BfACP3 Interfered With the Self-Ubiquitination of TRAF6

K63-linked polyubiquitination of TRAF6 is essential for NF-κB signaling (30). Such self-linked polyubiquitin chains serve as a scaffold to assemble the downstream signaling complex consisting of TAB2, NEMO, TAK1, and IKKs, which subsequently leads to IκB degradation and NF-κB activation (31). K48-linked polyubiquitination of TRAF6, however, might lead to protein degradation and hence negatively regulate the NF-κB pathway (32). We performed ubiquitination assays by transfecting 293T cells with Flag-tagged bfTRAF6 and HA-tagged wild-type or mutant ubiquitin (Ub, Ub-K63 and Ub-K48) in the presence or absence of bfACP3 proteins. We found that bfACP3 could significantly inhibit the ubiquitination of bfTRAF6, including both K63- and K48-ubiquitination (**Figure 6F**). This suggests that like amphioxus ACP2, bfACP3 might suppress TRAF6 functions by interfering with its ubiquitination.

### BfACP3 Used an Unknown Conserved Region to Interact With TRAF6

The amphioxus ACP2 could use its N-terminal non-ApeC region (**Figure 1A**) to interact with TRAF6, and putative TRAF6-binding motif could be found in this region (1). However, we did not find any TRAF6-binding motif in bfACP3, though it has an even longer N-terminal non-ApeC region (**Figure 1A**). We guessed that other sequences might be responsible for the interaction between bfACP3 and bfTRAF6. To determine which sequence of bfACP3 was used to recognize bfTRAF6, we constructed different truncated forms of bfACP3 (**Figure 6G**). Co-IP assays revealed that the 205-358aa region played the main function of interacting with TRAF6 (**Figure 6H**). This region is conserved among ACP1, ACP2, ACP3 and ACP5, located in the N-terminal non-ApeC region and adjacent to the ApeC domain (**Figure 1A** and **Supplementary Figure 3**). The identities of this region between different ACPs are modest (43-64%). It is rich in leucine but does not have a typical leucine-rich repeat (LRR). These results suggested that bfACP3 used an unknown conserved sequence in its N-terminal non-ApeC region to interact with bfTRAF6.

## Discussion and Conclusions

### ApeC Is a Novel Protein Domain Widely Distributed in Invertebrates

ApeC-containing proteins consist of a large protein family which are widely distributed in invertebrates, especially in invertebrates from aquatic and humid environments (2). ApeC-like domains were also found in bacteria, suggesting the ancient origins and broad distribution of ApeC (2). ApeC is a typical promiscuous domain like the IgSF domains and C-type lectin domains, capable of forming domain combinations with various domains. So far tens of different domain architectures have been documented in ACPs. Gene expression regulation and tissue distribution of some ACPs have been examined in different invertebrates, including sea urchins, sea cucumbers, corals, amphioxus, oysters and mussels, etc. (8–13, 33–36). Current data suggest that some ACPs may have an important role in embryogenesis, development and immune responses (5, 7–13, 33–41), but in most cases, the underlying mechanisms have not been investigated. In fact, most ACPs have not been functionally investigated.

Apextrin is the first ACP subfamily received detailed study. A typical apextrin has a signal peptide, an N-terminal membrane attack complex/perforin (MACPF) domain and a C-terminal ApeC domain, though the ApeC domain had not been recognized in early days. An apextrin from the sea urchin was found to be expressed in large quantities from fertilization to metamorphosis, and was proposed to be involved in the adhesion of apical cells and strengthening the outer layer of the embryo (6, 7), despite that the underlying molecular mechanism remains unclear. Later, apextrins have been found in at least five animal phyla (2).

### A Subfamily of Amphioxus ACPs Act as Pattern Recognition Proteins

An early study suggested that amphioxus ACP1 and ACP2 (both from *B. japonicum*) could function as pathogen-associated molecular pattern (PAMP) recognition proteins (1). In this study, we characterized another two amphioxus ACPs (ACP3 and ACP5 from *B. floridae*). ACP3/5 and ACP1/2 may have related functions, because the similar domain architecture and the close phylogenetic relation suggest that they belong to the same subfamily (**Figures 1A, B** **)**. But they also have distinct differences. ACP3 and ACP5 only share 40-50% sequence identities with ACP1 and ACP2. ACP3 and ACP5 have longer N-terminal sequences than ACP1 and ACP2. ACP5 even has an additional CUB domain, making it more akin to extracellular matrix proteins or humoral proteins. In the complement proteases, CUB domains can mediate dimerization and binding to collagen regions of partner proteins (28).

ACP1 and ACP2 are highly expressed in several mucosal and non-mucosal tissues, with the highest concentration in the gill and skin. While ACP3 and ACP5 have a more limited tissue distribution, predominantly expressed in the gut. This suggests that though they all function in mucosal tissues, ACP3/5 and ACP1/2 specialize in different niches. In line with this, they have different microbial binding spectrum. Recombinant ACP1 and ACP2 were shown to bind Gram-positive bacteria (1), while recombinant ACP5 exhibited binding capacity with yeasts, Gram-positive and Gram-negative bacteria (**Figure 3**). Recombinant ACP3 had a similar spectrum with ACP5, but its binding strength to Gram-negative bacteria was much weaker (**Figure 3**). Despite all these, no recombinant proteins (ACP1, ACP2, ACP3 and ACP5) showed killing or inhibitive effects on microbes (**Figure 4**), suggesting that they more likely act as mere lectins.

The bacterial cell wall component PGN and its basic active motif MDP have been identified as binding ligands for recombinant ACP1 and ACP2 (1). Though recombinant ACP3 and ACP5 were also found to recognize PGN, they displayed a more selective specificity. They only bound to the Lys-type PGN of Gram-positive bacteria, and had no detectable affinity for three basic motifs of PGN, including MDP, NAM and NAG (**Figure 5**). Moreover, recombinant ACP3 and ACP5 were also found to bind yeasts and some Gram-negative bacteria (**Figures 3C, D** **)**, but we failed to determined what ligands they used to recognize yeasts and Gram-negative bacteria (**Figures 3** **–** **5**). There are several possibilities related to the observed binding specificities: ACP3/5 have different structural characteristics than ACP1/2, the recombinant ACP3/5 might not preserve all the properties of the native proteins, and the purified microbial cell wall components might lose their native conformation.

### This Subfamily of Amphioxus ACPs Regulate the TRAF6-NF-κB Pathway

In addition to being an extracellular effector, intracellular ACP2 was found capable of modulating the TRAF6-NF-κB pathway, possibly as a feedback inhibitory mechanism to control the magnitude of the immune response (1). It could interact with TRAF6 and prevent TRAF6 from self-ubiquitination and hence from activating NF-κB. Here we found that ACP3 also has this intracellular function, to suppress the TRAF6 induced NF-κB activation by binding TRAF6 and interfering with the ubiquitination of TRAF6 (**Figure 6**). Here we further pinpointed the region responsible for the TRAF6 binding, and this region is adjacent to the ApeC domain and relatively conserved in ACP1, 2, 3 and 5 (**Figure 1A** and **Supplementary Figure 3**).

## Concluding Remarks

Taken together, this study provides a second but different line of mechanistic evidence for the immune role of ACPs in amphioxus. This study, together with the previous studies (1, 15, 38), defines a novel subfamily of ACPs, which share a conserved structure and have similar yet diversified molecular functions. The members of this ACP subfamily adopt a dual-functional mode: the ApeC domain serves the lectin role, while a conserved unknown region serves as a signal transduction regulator for the TRAF6-NF-κB pathway. This work broadens our understanding of the ACP functions and may facilitate further research efforts on the role of ACPs in other animal clades.

## Data Availability Statement

The original contributions presented in the study are included in the article/**Supplementary Material**. Further inquiries can be directed to the corresponding authors.

## Author Contributions

JL, SH and AX designed the study. JL and YL performed the experiments. JL, YL and SH analyzed the data. ZF, SheC, XY, ZY, GH, SL, MD, ShaC, and HZ also conducted some experiments, provided reagents or analyzed the data. JL and SH drafted the manuscript. AX revised the manuscript. All authors contributed to the article and approved the submitted version.

## Funding

This work was supported by NNSF Projects (31872595 & 31722052 & 31971107), National Key R&D Program of China (2018YFD0900503), Marine S&T Fund of Shandong Province for Pilot National Laboratory for Marine Science and Technology (2018SDKJ0302-2), Project supported by Innovation Group Project of Southern Marine Science and Engineering Guangdong Laboratory (Zhuhai) (No. 311021006), and projects from Guangdong and Guangzhou (2021A1515012380 & 2020B1212060031).

## Conflict of Interest

The authors declare that the research was conducted in the absence of any commercial or financial relationships that could be construed as a potential conflict of interest.

## Publisher’s Note

All claims expressed in this article are solely those of the authors and do not necessarily represent those of their affiliated organizations, or those of the publisher, the editors and the reviewers. Any product that may be evaluated in this article, or claim that may be made by its manufacturer, is not guaranteed or endorsed by the publisher.
