# Supplementary material for: Two Amphioxus ApeC-Containing Proteins Bind to Microbes and Inhibit the TRAF6 Pathway
Source: Front Immunol. 2021 Jul 30;12:715245. doi: 10.3389/fimmu.2021.715245 (PMC8361754; doi:10.3389/fimmu.2021.715245)
Supplement: Supplementary file 1 [file DataSheet_1.docx]

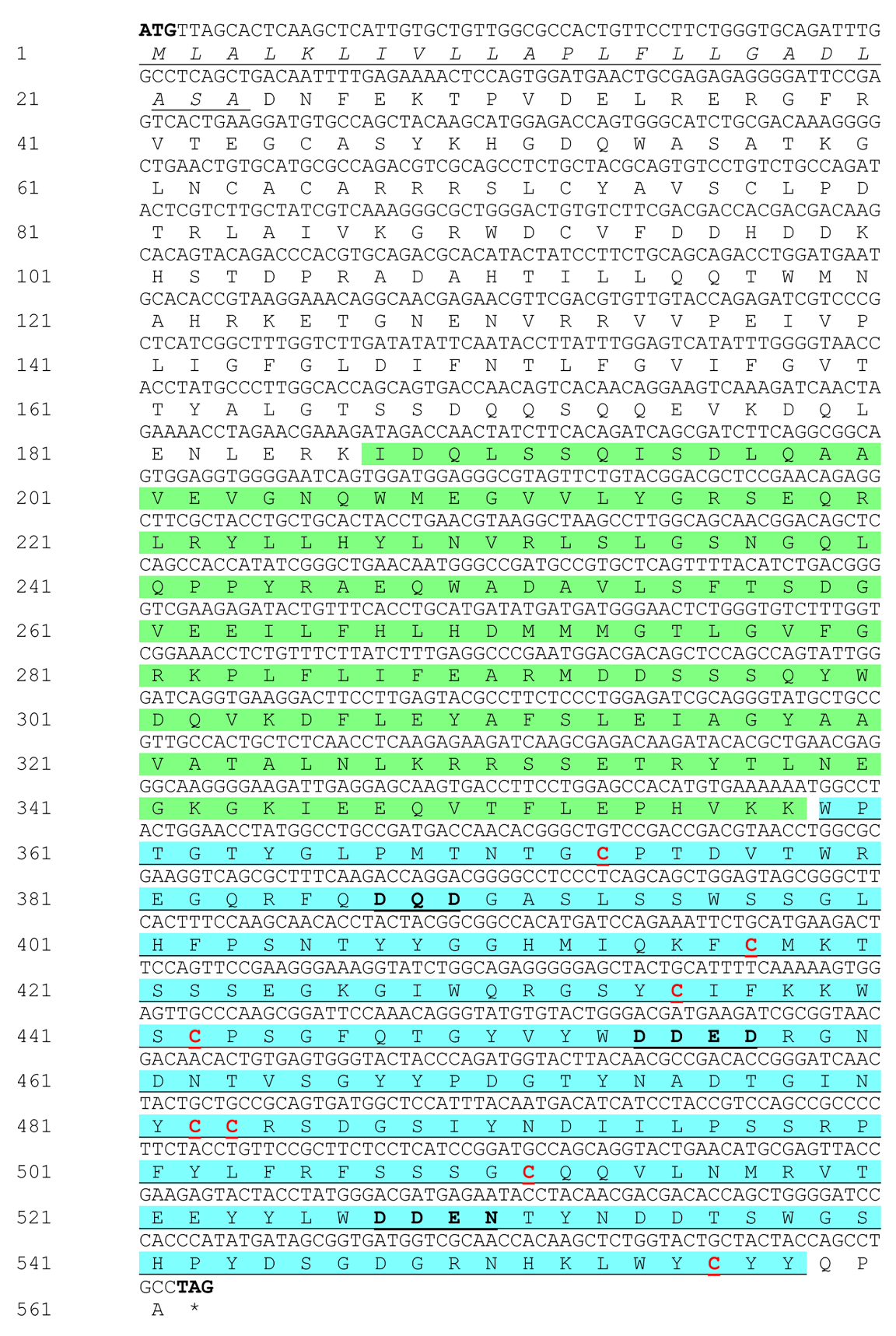


**Supplementary Figure 1.** Nucleotide and deduced amino acid sequences of bfACP3. The start codon (ATG) and the stop codon (TAA) are in bold. The signal peptide and the ApeC domain predicted by SMART are underlined, highlighted in italics and blue background, respectively. The unknown conserved region is marked in green. The eight conserved Cysteine residues are bold and red, and the three relatively conserved DXED motifs are bold.


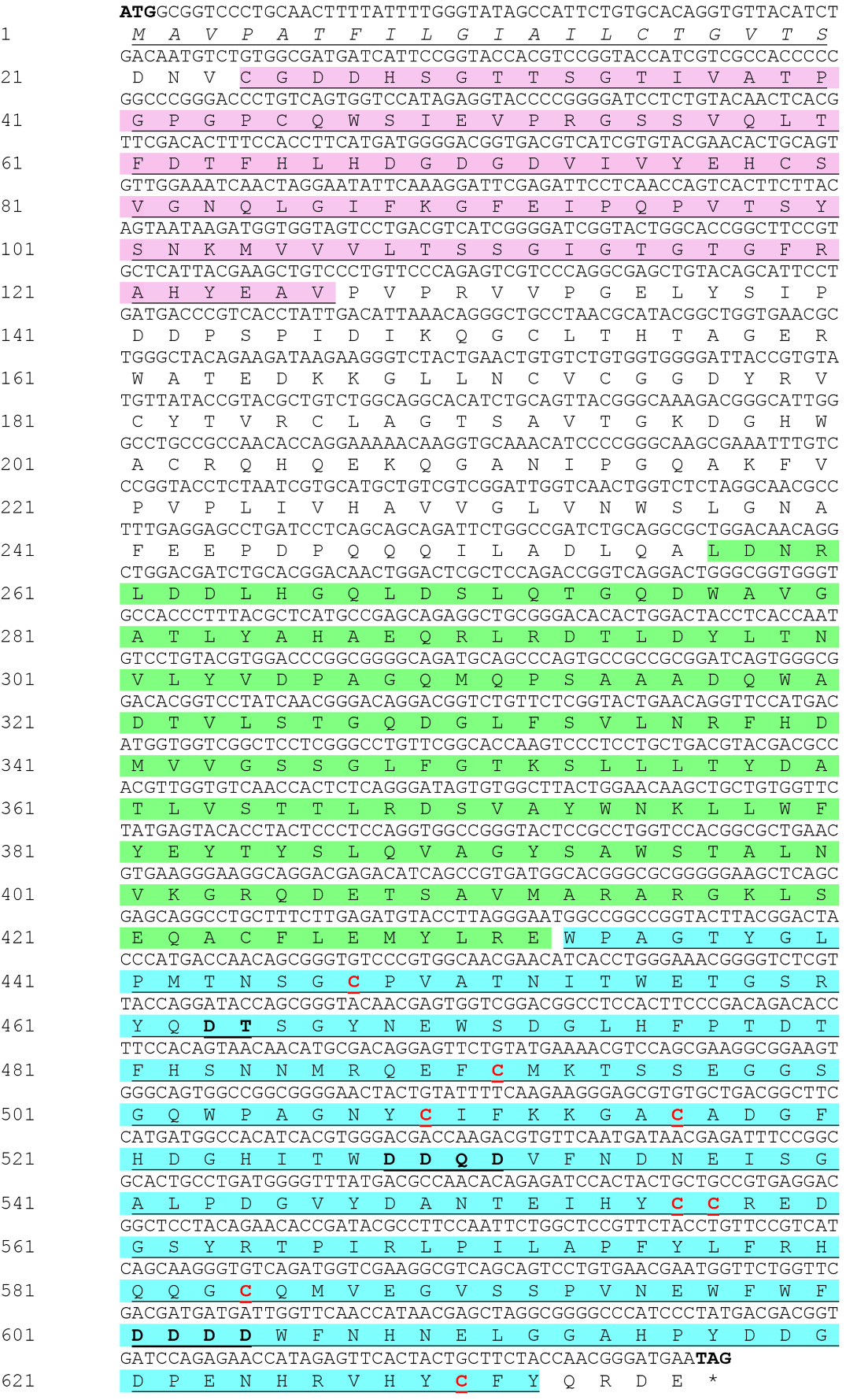


**Supplementary Figure 2.** Nucleotide and deduced amino acid sequences of bfACP5. The start codon (ATG) and the stop codon (TAA) are in bold. The signal peptide, the CUB domain and the ApeC domain predicted by SMART are underlined, highlighted in italics, pink and blue background, respectively. The unknown conserved region is marked in green. The eight conserved Cysteine residues are bold and red, and the three mutated DXED motifs are bold.


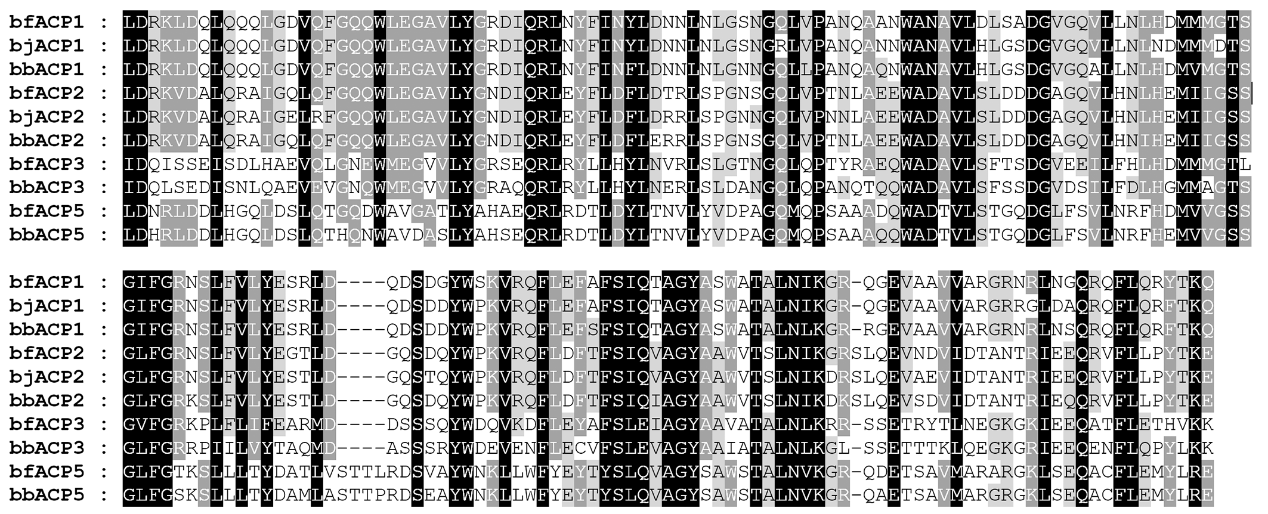


**Supplementary Figure 3.** Multiple alignment of the unknown conserved region of amphioxus ACPs. bj, *Branchiostoma japonicum*; bf, *Branchiostoma floridae*; bb, *Branchiostoma belcheri*.


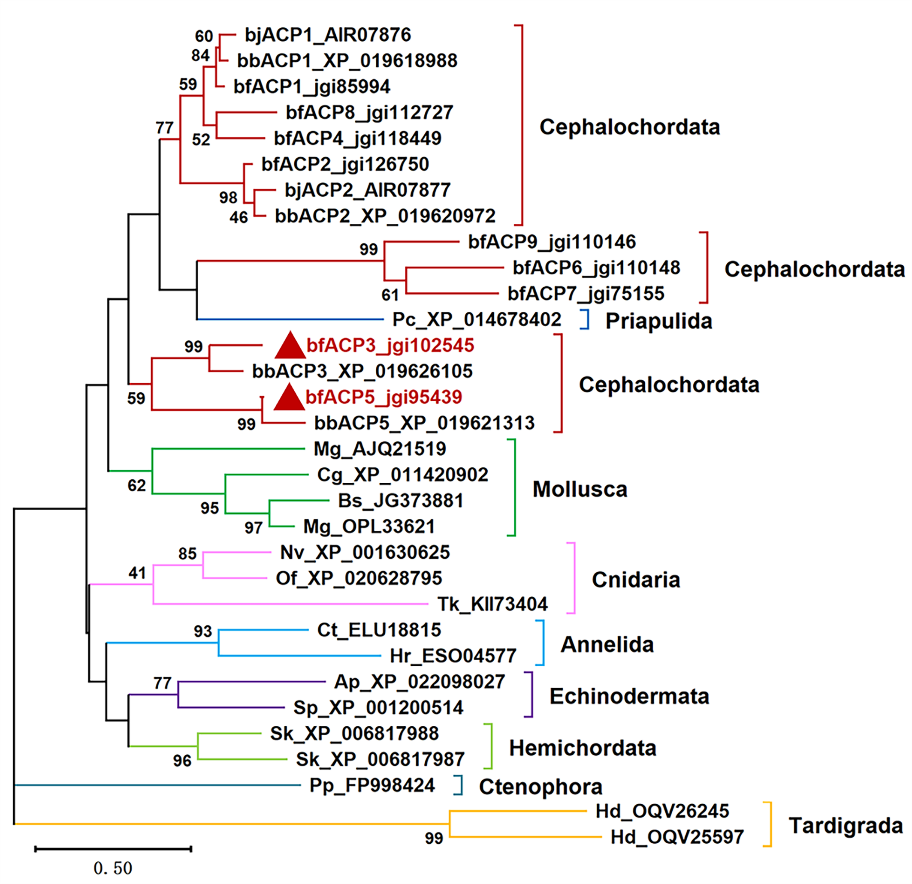


**Supplementary Figure 4.** The Maximum Likelihood tree of representative ACPs from different aquatic animal phyla based on the sequence of ApeC domains. Evolutionary analyses were conducted in MEGA-X with WAG model, Gamma distribution of rates across sites model and 1000 bootstrap tests. The percentage of trees in which the associated taxa clustered together is shown next to the branches. The tree is drawn to scale, with branch lengths measured in the number of substitutions per site.


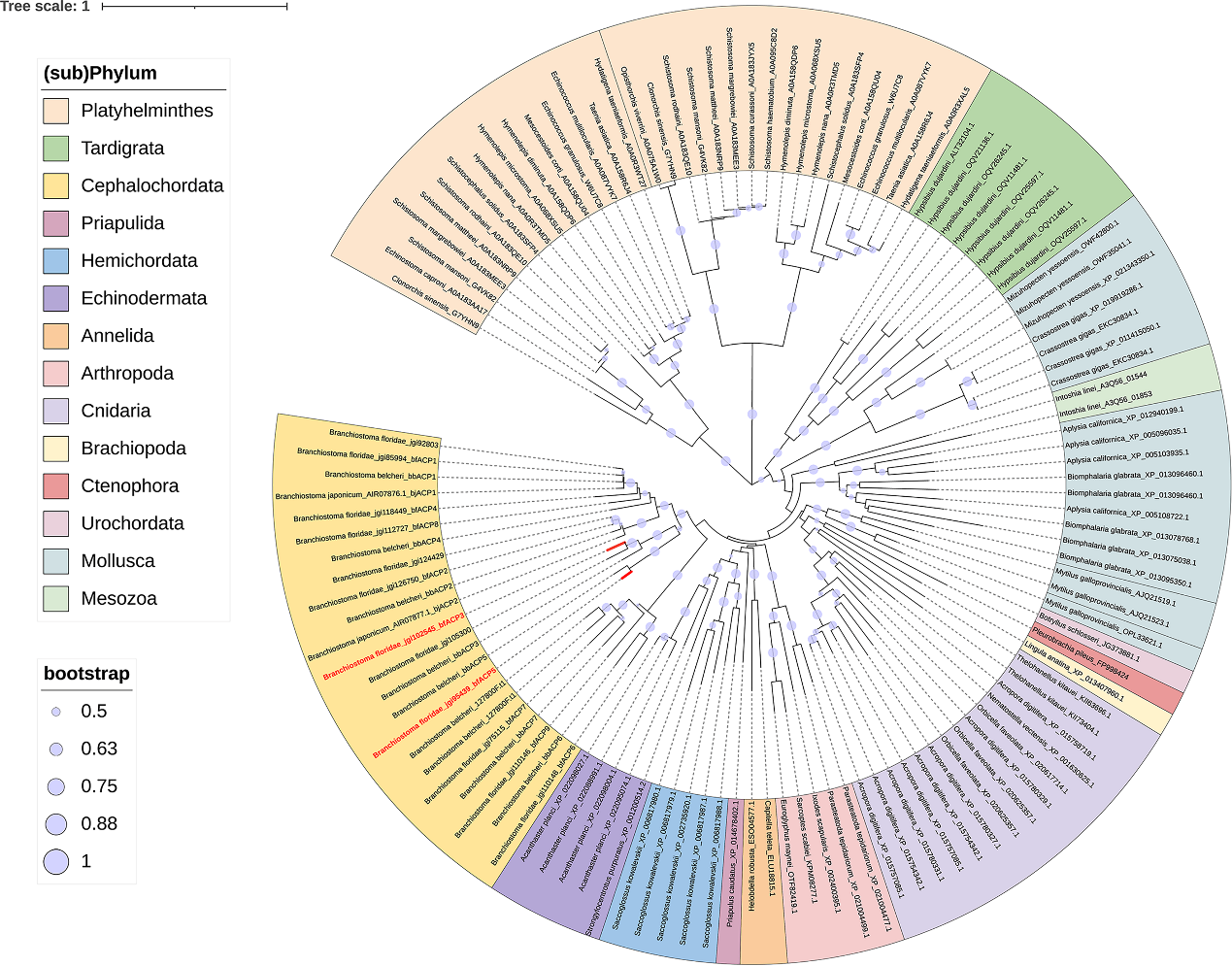


**Supplementary Figure 5.** An expanded phylogenetic analysis of representative ACPs from animal phyla. This tree is a more detailed version of the tree shown in **Figure 1B**. The tree was constructed using the neighbor-joining method in MEGA-X with the JTT matrix-based method, 1000 bootstrap tests and handling gaps by pairwise deletion, and was optimized on iTOL website.


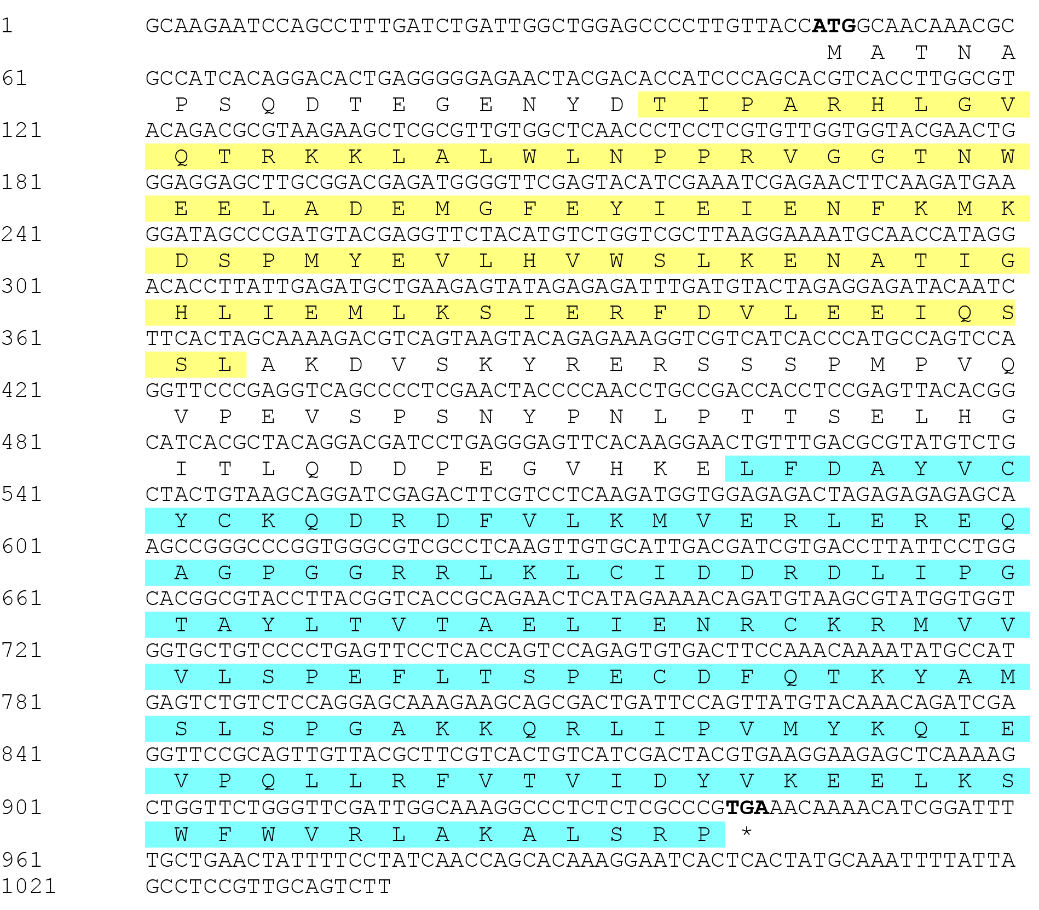


**Supplementary Figure 6.** Nucleotide and deduced amino acid sequences of bfMyD88. The start codon (ATG) and the stop codon (TAA) are in bold. The DEATH domain and TIR domain predicted by SMART are highlighted in yellow and blue background, respectively.


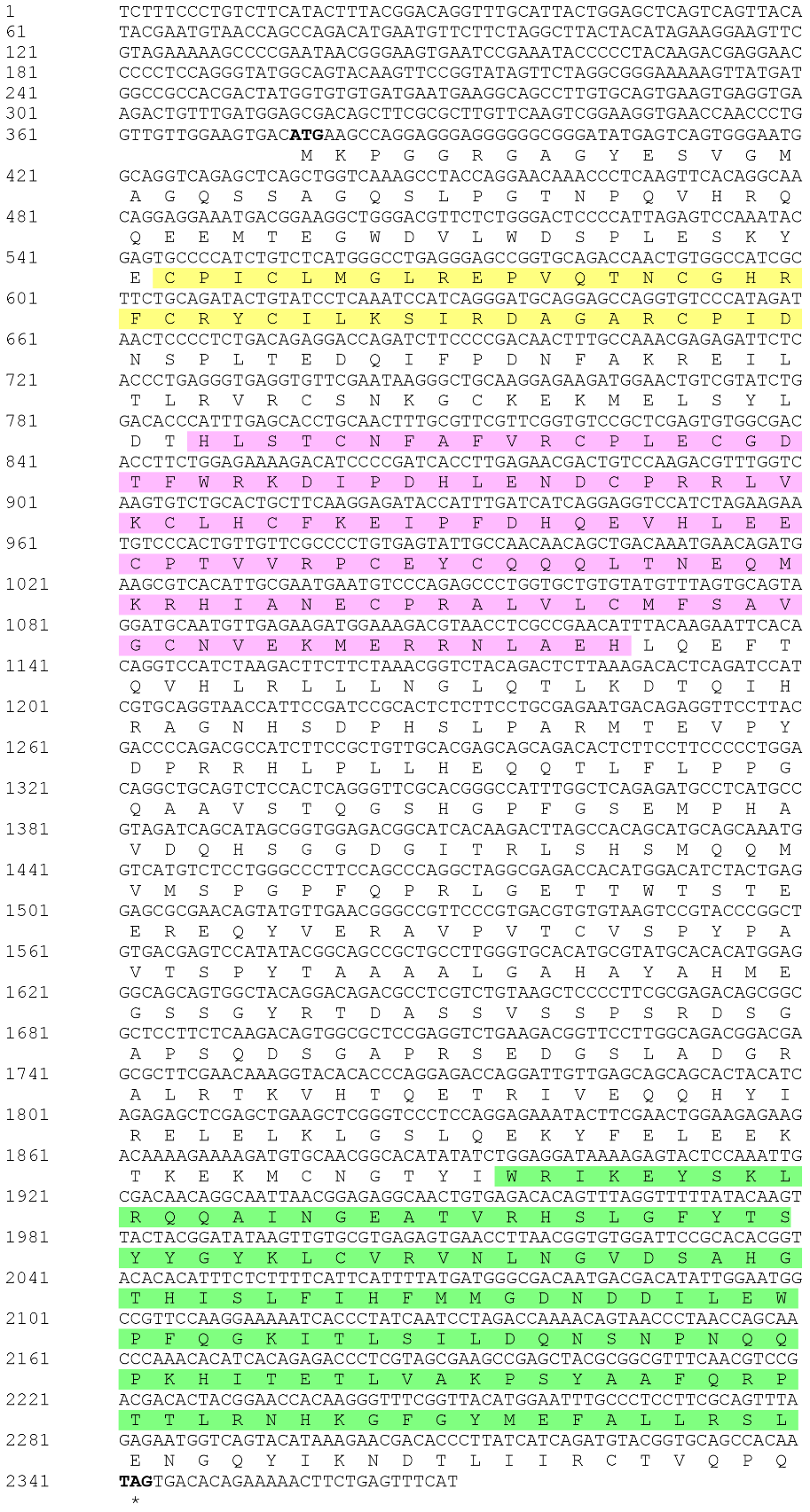


**Supplementary Figure 7.** Nucleotide and deduced amino acid sequences of bfTRAF6. The start codon (ATG) and the stop codon (TAA) are in bold. The RING domain, zf-TRAF domain and MATH domain predicted by SMART are highlighted in yellow, pink and green background, respectively.
